# Resting-state functional MRI demonstrates brain network reorganization in neuromyelitis optica spectrum disorder (NMOSD)

**DOI:** 10.1371/journal.pone.0211465

**Published:** 2019-01-29

**Authors:** Kévin Bigaut, Sophie Achard, Céline Hemmert, Seyyid Baloglu, Laurent Kremer, Nicolas Collongues, Jérôme De Sèze, Stéphane Kremer

**Affiliations:** 1 Department of neurology, Hôpitaux Universitaires de Strasbourg, Strasbourg, France; 2 Centre National de la Recherche Scientifique, Grenoble Image Parole Signal Automatique, Grenoble, France; 3 Department of radiology, Groupe Hospitalier Régional Mulhouse Sud-Alsace, Mulhouse, France; 4 Department of radiology, Hôpitaux Universitaires de Strasbourg, Strasbourg, France; Inria - ICM, Paris, FRANCE

## Abstract

**Background:**

The relation between brain functional connectivity of patients with neuromyelitis optica spectrum disorder (NMOSD) and the degree of disability remains unclear.

**Objective:**

Compare brain functional connectivity of patients with NMOSD to healthy subjects in resting-state functional MRI (rs-fMRI).

**Methods:**

We compared the rs-fMRI connectivity in 12 NMOSD patients with 20 healthy subjects matched for age and sex. Graph theory analysis was used to quantify the role of each node using a set of metrics: degree, global efficiency, clustering and modularity. To summarize the abnormal connectivity profile of brain regions in patients compared to healthy subjects, we defined a hub disruption index κ.

**Results:**

Concerning the global organization of networks in NMOSD, a small-world topology was preserved without significant modification concerning all average metrics. However, visual networks and the sensorimotor network showed decreased connectivity with high interindividual variability. The hub disruption index κ was correlated to the Expanded Disability Status Scale (EDSS).

**Conclusion:**

These results demonstrate a correlation between disability according to the EDSS and neuronal reorganization using the rs-fMRI graph methodology. The conservation of a normal global topological structure despite local modifications in functional connectivity seems to show brain plasticity in response to the disability.

## Introduction

Neuromyelitis optica spectrum disorder (NMOSD) is an inflammatory disease of the central nervous system characterized by severe relapses in regions with high aquaporin 4 (AQP4) expression such as the optic nerves, the spinal cord and specific brain areas.

In 43–70% of patients with NMOSD, brain MRI abnormalities have been reported mainly in regions with high AQP4 expression, in corticospinal tracts and also in deep white matter with nonspecific lesions [1]. However, using diffusion tensor imaging (DTI), normal-appearing white matter abnormalities have been described, mostly in optic radiations and corticospinal tracts [2]. These abnormalities in DTI are also found in the normal-appearing white-matter of multiple sclerosis (MS) patients but are not restricted to corticospinal tracts [3]. In NMOSD and MS, an association between white matter diffusion changes and disability (evaluated by the Expanded Disability Status Scale, EDSS) has been reported [3,4].

In rs-fMRI, previous studies for MS patients reported functional connectivity changes in a limited number of brain regions but keeping a global brain integrity [5,6]. Similar results with fMRI demonstrated a correlation between modification in the functional connectivity of brain regions and disability in MS but not yet for NMOSD [7–10].

Graph theory is a mathematical model used to describe the brain network topology as graphs of regional cortical and subcortical nodes. This approach analyzes the interactions between several regions by evaluating the functional organization of nodes, the strength of the interactions, and the efficiency of information processing [11,12]. rs-fMRI studies demonstrated that the human brain network has a small-world topology with highly connected hub nodes [13]. With this organization, the brain can process specialized and integrated information. A small-world topology is conserved in comatose patients and other neurological diseases [11,14].

Our hypothesis is that the functional connectivity of brain regions in patients with NMOSD are disturbed compared to healthy subjects and these modifications are correlated with the disability. The objective of this study was to compare the functional connectivity of NMOSD patients to healthy subjects in rs-fMRI using graph theory.

## Materials and methods

Twelve patients with NMOSD defined according to the 2015 international consensus diagnostic criteria were recruited from the Strasbourg University Hospital [15]. Inclusion criteria were patients with defined NMOSD who were relapse-free. Healthy subjects had no personal history of neurological or psychiatric disease and had taken no medication, alcohol or drugs. The duration of the disease, the degree of the disability as assessed by the EDSS and presence of anti-AQP4 antibodies were collected.

The study protocol was approved by the local ethics review board (Comité de Protection des Personnes) and all participants gave written informed consent before participation.

MRI was performed on a 1.5T MRI (Avanto, Siemens, Erlangen, Germany). Anatomical images were acquired using a high-resolution three-dimensional (3D) T1 and for functional images (patients with open eyes) using gradient-echo echo planar imaging (EPI) with blood oxygen level-dependent (BOLD) contrast with the following parameters: TR = 3 s, TE = 50 ms, 4×4×4 mm^3^ isotropic voxel size, 405 images and 32 axial slices on the whole cortex.

Resting-state fMRI data were corrected with anatomical images then normalized with the template Colin27 and analyzed with the SPM8 program [16]. This technique produces subdivided images (template automated labeling) in 90 brain regions (nodes) [17].

For each node, we computed topological metrics: degree (the number of connections per node), global efficiency (link to the shortest path between nodes), clustering (quantification of the connections among the neighborhoods of a node) and modularity (measurement of the possibility of dividing the network into subgroups of nodes). The overall average of these metrics was estimated in each network, but also explored at the nodal level.

To summarize the abnormal profile of nodal connectivity in patients compared to healthy subjects, we defined the hub disruption index κ [14]. It is computed by fitting a straight line (linear regression) to a scatterplot of the nodal property of interest, such as degree, in an individual participant minus the same nodal property on average over all of the healthy volunteers versus the mean nodal property in the healthy group and is defined by the slope of this straight line. A high κ value associated with a conservative average value is related to significant reorganization, where hubs become non-hubs and vice-versa.

For the statistical analysis, we regressed metrics with EDSS using a linear model. For each test, we performed an analysis of variance (ANOVA) with the *p*-value. Global and nodal statistics were compared between groups using *t*-tests or permutation tests. No multiple comparisons were applied, and *p*<0.01 was considered significant for descriptive purposes.

## Results

Twelve NMOSD patients (six males and six females) were included with a mean age of 45.6 years (range, 25–66 years), a mean disease duration of 10.5 years, a mean EDSS of 3.5, and seven patients had anti-AQP4 antibodies (Table 1). Twenty healthy volunteers (11 males and nine females; range, 25–51 years) were included.

**Table 1:** Demographic, clinical and biological data of the patients included.

	Sex	Age, year	EDSS	Duration of disease, year	anti-AQP4 antibody
**Patient 1**	female	41	2	6	Yes
**Patient 2**	female	66	4	12	Yes
**Patient 3**	male	46	8	9	No
**Patient 4**	male	49	7	5	No
**Patient 5**	female	30	2	6	Yes
**Patient 6**	female	49	0	34	Yes
**Patient 7**	female	57	2	17	Yes
**Patient 8**	male	35	3	6	No
**Patient 9**	male	51	6	5	Yes
**Patient 10**	female	25	3	3	No
**Patient 11**	male	48	3	13	No
**Patient 12**	male	51	2	10	Yes

EDSS, Expanded Disability Status Scale; AQP4, aquaporin 4.

**Fig 1:**
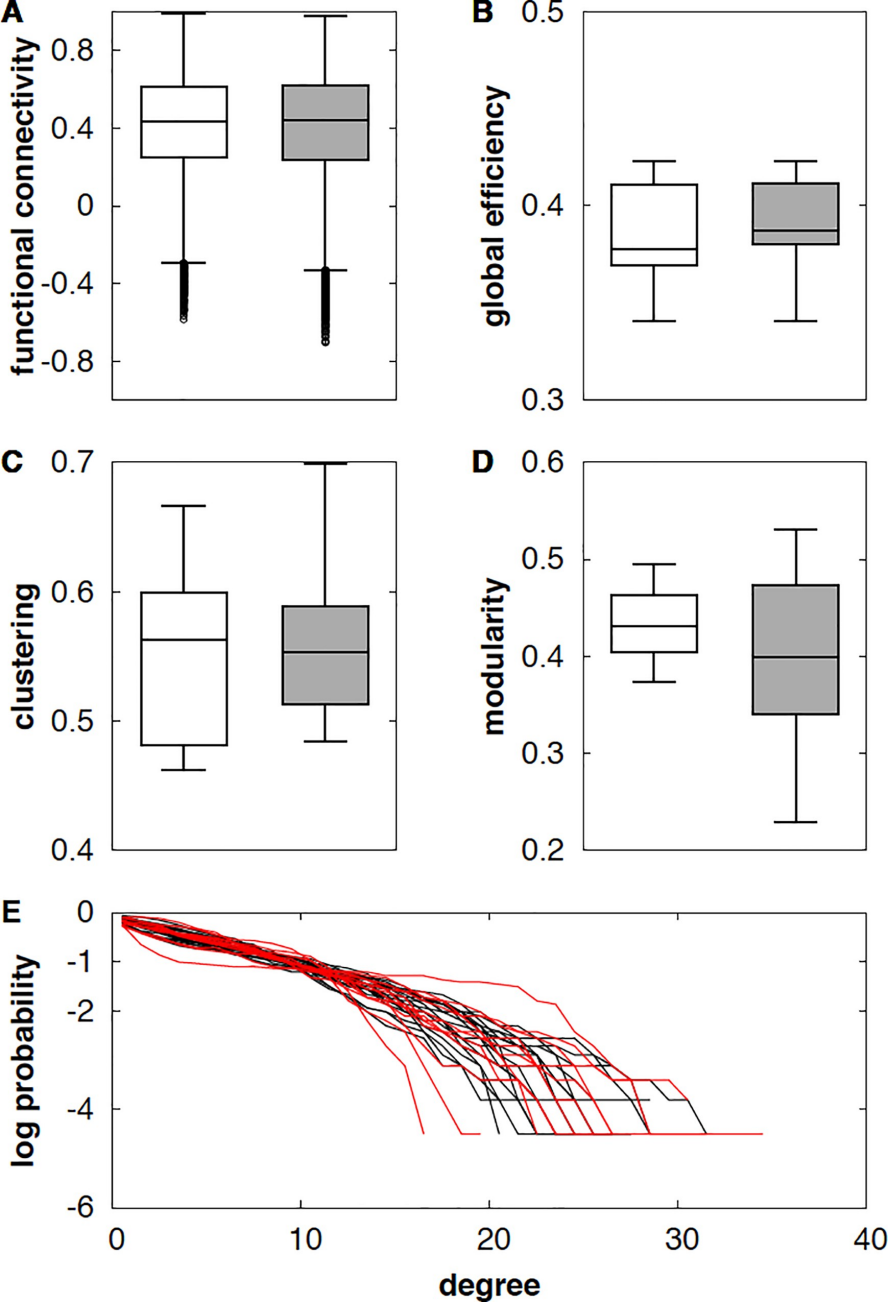
Preserved small-world topological organization of NMOSD patients. Average metrics are extracted for each brain network for healthy subjects and patients ((A) functional connectivity, gathering information on correlations; (B) global efficiency, related to the shortest path; (C) clustering, quantifying connections of the neighborhood; (D) modularity, measuring the possibility of dividing the network into subgroups of nodes); (E) degree distribution, the probability distribution of the degree of a node in the network (patients in red and healthy volunteers in black). No statistical differences were observed between NMOSD patients (grey) and healthy subjects (white) for all metrics used. This shows that the small-world topology is preserved for NMOSD patients despite the disability that is already present on clinical assessment of the patients. Abbreviations. NMOSD, neuromyelitis optica spectrum disorder.

At a global level, there was no significant difference in mean values for global efficiency, clustering, modularity and degree between the NMOSD group and the control group (Fig 1). The small-world topology, as described in Watts and Strogatz and revealed in the brain graph topology of healthy subjects, was characterized by a short minimum path length producing an efficient graph in terms of transmission of information and by high clustering reflecting higher resilience to attack because neighborhoods of nodes are well connected [18]. Despite the pathology, the functional connectivity network of NMOSD patients maintained a small-world topology with high global efficiency and strong clustering, as in healthy subjects.

**Fig 2:**
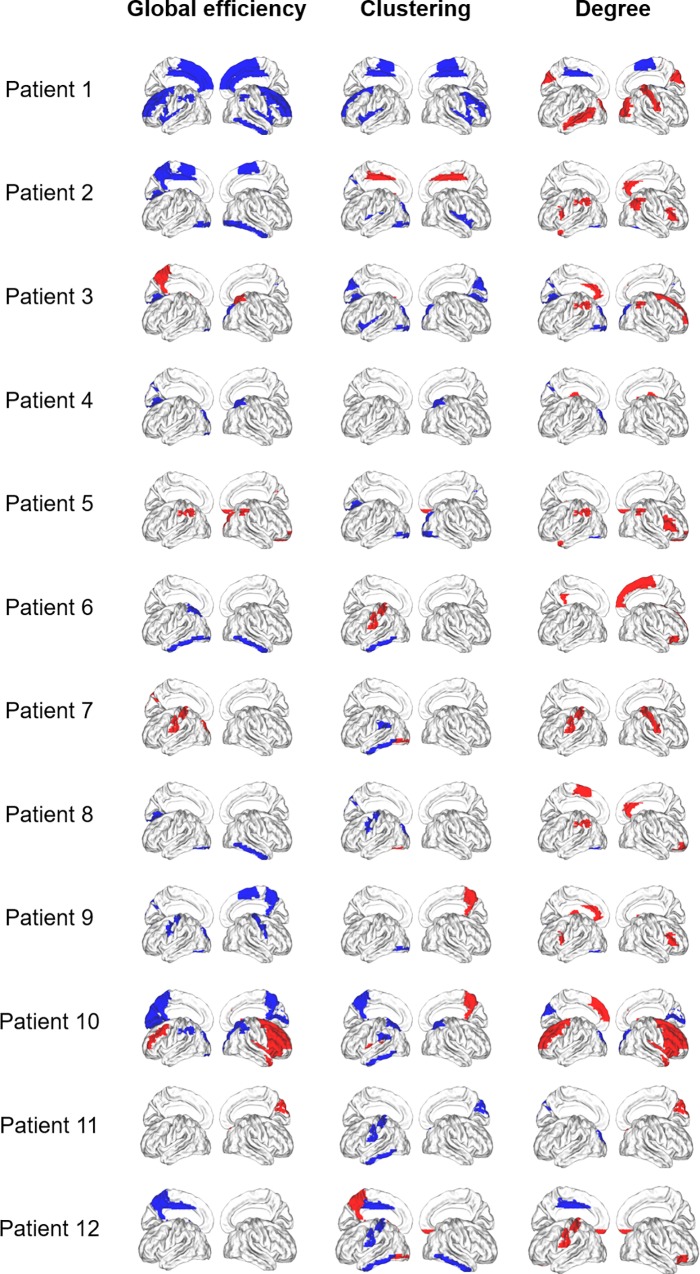
Disconnected and overconnected nodes in NMOSD patients. Cortical surface representation of the difference in mean global efficiency, clustering, and degree between patient and volunteer groups (in red significantly increased parameter in NMOSD patients compared with healthy subjects, and in blue significantly decreased parameter in NMOSD patients).

At a regional level, there were significant differences (*p*<0.01) in the functional connectivity of some brain regions between NMOSD patients compared to healthy participants with a high interindividual variability for global efficiency, clustering and degree (Fig 2). There was no correlation between regional values and EDSS scores.

**Fig 3:**
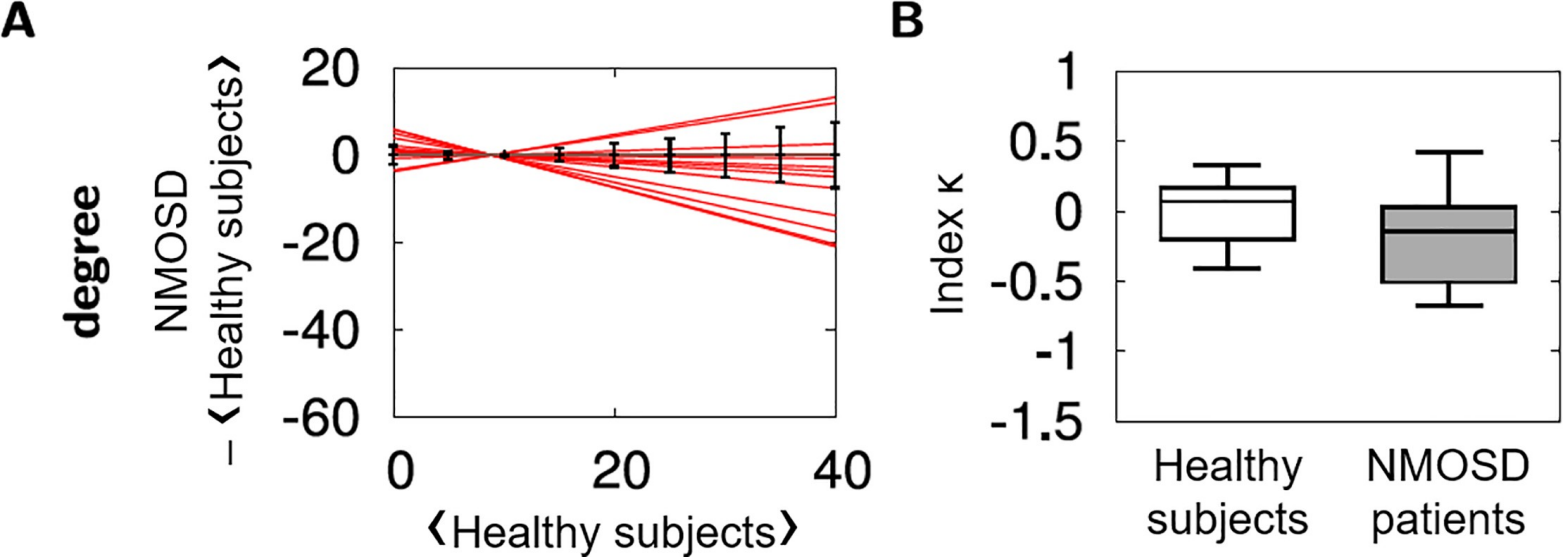
Comparison of nodal degree and hub disruption index κ between NMOSD patients and healthy subjects. (A) Estimated linear regression for each NMOSD patients (red lines) computed from a scatterplot of the nodal degree in an individual participant minus the same nodal property on average over all of the healthy volunteers versus the mean nodal property in the healthy group. Error bars in black reflect standard deviation calculated from the nodal degree in healthy subjects. (B) Boxplots of hub disruption index κ in the control group (white) and the NMOSD group (grey). There was no difference between the two groups.

**Fig 4:**
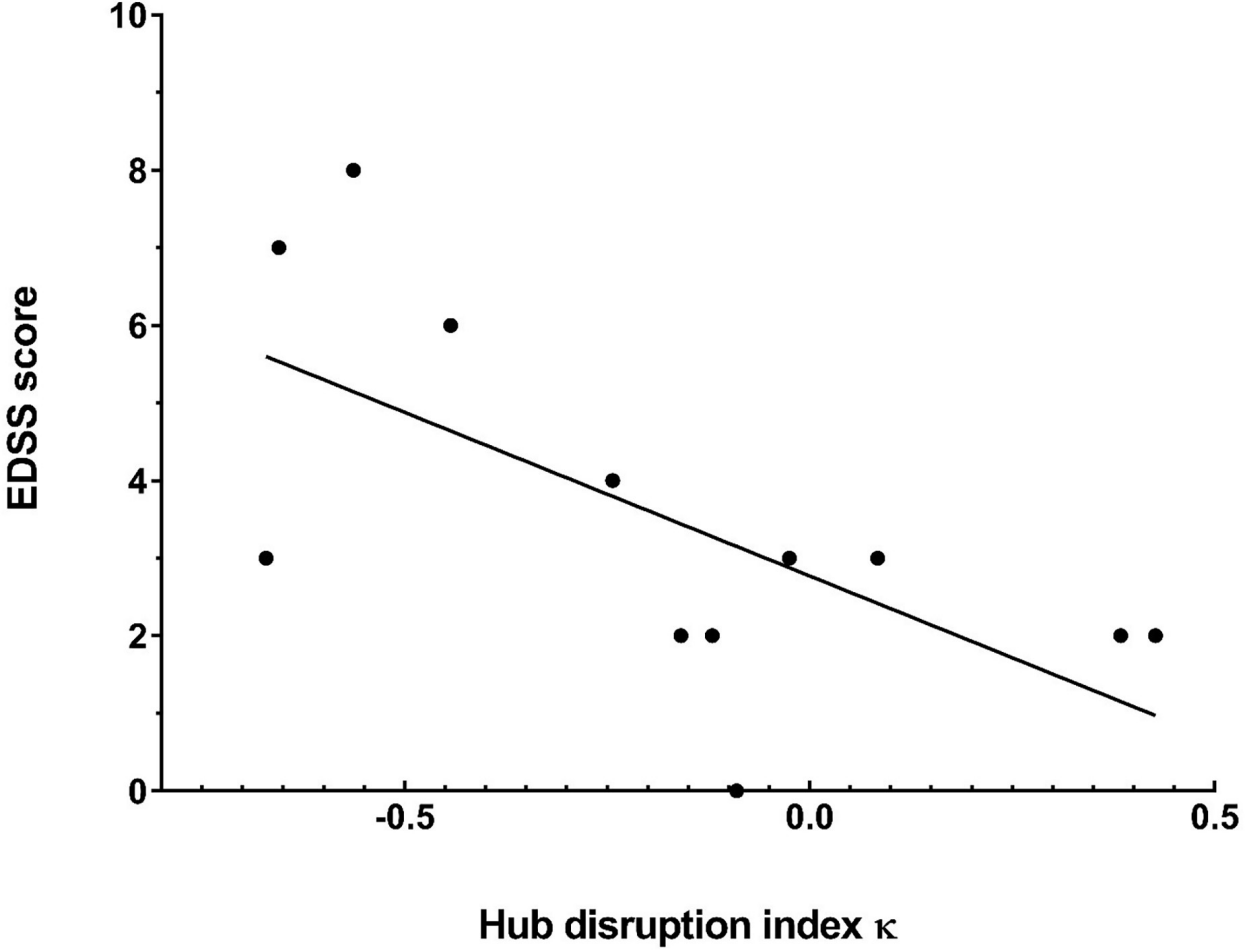
Brain network reorganization is greater when the disability as measured with EDSS is higher. The hub disruption index κ and the EDSS score are presented for each NMOSD patient. The black line corresponds to the estimated regression between the global reorganization index and the EDSS score. A hub disruption index of 0 corresponds to a network similar to healthy participants. The hub disruption index κ is correlated with the EDSS score. The correlation shows that reorganization of brain networks is closely related to the increase in disability for these patients. This may reflect a compensation mechanism that is active throughout the disease. Abbreviations. EDSS, Expanded Disability Status Scale.

The functional connectivity was modified in many brain regions in NMOSD patients compared to healthy subjects, in particular for the degree. The hub disruption index κ of each NMOSD patients was calculated from the linear regression based on the differences in the degree between each NMOSD patients and the healthy group (Fig 3A and Table 2). Although differences were observed between an individual NMOSD patient and the healthy group, there was no significant difference between the averages of κ in NMOSD patients compared to healthy participants attesting a high interindividual variability (Fig 3B). However, there was a significant correlation (*p* = 0.006) between κ and the EDSS (Fig 4). The higher the disability evaluated by EDSS, the more κ deviates from 0.

**Table 2:** The hub disruption index κ and EDSS score for each NMOSD patients.

NMOSD patients	Hub disruption index κ value	EDSS score
**Patient 1**	0,427	2
**Patient 2**	-0,243	4
**Patient 3**	-0,563	8
**Patient 4**	-0,655	7
**Patient 5**	-0,159	2
**Patient 6**	-0,09	0
**Patient 7**	0,384	2
**Patient 8**	-0,025	3
**Patient 9**	-0,443	6
**Patient 10**	-0,671	3
**Patient 11**	0,084	3
**Patient 12**	-0,12	2

## Discussion

The application of graph theoretical analysis to fMRI provides a global view of the connectivity network for each individual and quantifies the role of each brain region within the network [11]. Thus, it is possible to identify decreased or increased connectivity in cortical regions in NMOSD patients.

The structural cortical networks in NMOSD demonstrated small-world organization as in MS, organized into modules with hubs (strongly connected nodes, high degree) as in healthy subjects [11].

The results show that there are many brain regions where regional functional connectivity is modified in NMOSD patients compared to controls with a high interindividual variability. The main regions are involved in the visual network and the sensorimotor network. Our data differ from other studies because they only show modifications for a few regions. These results are consistent with preferential involvement of the optic nerves and spinal cord in NMOSD and confirm DTI studies [4]. The axonal lesions could lead to a decrease in the functional connectivity of the cerebral regions related by Wallerian degeneration [2].

To objectify reorganization of the functional networks, we defined a hub disruption index κ, which has already been used in studies in comatose patients, patients with stroke, lateralized focal epilepsies and clinically isolated syndrome (CIS) but never in patients with NMOSD [14,19–21]. These studies demonstrated a reorganization of brain networks with both disconnected and overconnected nodes in patients and a reduced hub disruption index κ in comparison with healthy subjects. In patients with CIS, there was any correlation between κ and the EDSS score or the patient’s lesion load [21]. In our study, κ in NMOSD group was not reduced in comparison to healthy group probably because of an important variability in the symptomatology between NMOSD patients. However, κ was correlated with the EDSS score. Therefore, local modifications of connectivity could lead to increased neuronal reorganization while preserving a normal global structure, which supports the idea of cerebral plasticity in response to disability as demonstrated in patients with RRMS or CIS [10,21,22]. The neuronal reorganization implemented to balance local modifications probably differs from one patient to another, which could explain the high interindividual variability of the data.

The main limitation of this study is the low number of patients increasingly limiting the probability of finding a significant variation in functional connectivity. The low number of patients is explained by the low prevalence of NMOSD. Secondly, these results are heterogeneous, with considerable differences in functional connectivity between patients.

In summary, we have shown that there is a significant correlation between disability measured by EDSS and network reorganization. These results suggest that network reorganization may highlight cerebral plasticity in response to clinical disability. The analysis of brain network reorganization by rs-fMRI needs to be investigated further in longitudinal studies.
